# Development of a dynamic blood vessel phantom for evaluation of moving images

**DOI:** 10.1002/acm2.12775

**Published:** 2019-11-28

**Authors:** Miwa Osawa, Keisuke Kondo, Sato Hisaya, Kyoichi Kato

**Affiliations:** ^1^ Graduate School of Medical Health Sciences Komazawa University Setagaya‐ku Tokyo Japan; ^2^ Radiation Technology Department Showa University Fujigaoka Hospital Aoba‐ku, Yokohama city Kanagawa Japan; ^3^ Graduate School of Health Sciences Showa University Shinagawa‐ku Tokyo Japan

**Keywords:** angiography, digital fluoroscopy, image analysis

## Abstract

In coronary angiography (CAG) and percutaneous coronary intervention (PCI), it is important for radiological technologists to optimize the balance between radiation dose and image quality for physicians to be able to perform CAG and PCI most effectively. Evaluation of image processing is necessary to ensure that technologists can optimally adjust image quality for clinical use to the extent that physicians require. However, few phantoms are available for evaluating fluoroscopic image processing, and this makes it necessary to adjust image quality in clinical settings while utilizing the image processing according to the manufacturer’s recommendations. In this study, we developed a dynamic phantom that mimics a pulsating coronary artery for use in image quality analysis of moving images. We also examined whether processed images (image lag) can be physically analyzed. Two issues require special attention in creating a new phantom: establishing the exact position of the simulated blood vessel in the phantom, and providing good reproducibility. The study used the rotational motion of a disk to generate images, with a circular simulated blood vessel on the rotating acrylic disk, with the center of the simulated blood vessel shifted from the center of the acrylic disk. This enabled the reproduction of translational motion of the simulated blood vessel. As a result, because this phantom has signals and afterimages at the same position (of a simulated blood vessel), a quantitative evaluation of the afterimages became possible. In the evaluation of the image processing with the angiograph, it was shown that evaluations of image lag which are typically performed in clinical settings can be performed with the dynamic blood vessel phantom.

## 1 INTRODUCTION

In coronary angiography (CAG) and percutaneous coronary intervention (PCI), blurring of the constantly pulsing coronary artery may interfere with diagnosis and treatment. For this reason, it is important that technologists can adjust image quality optimally with X‐ray fluoroscopy, for clinical use to the extent that physicians require.

There are cases where the quality of images does not meet the standards required by the physicians performing CAG and PCI, arising as a result of the processing of the moving images (fluoroscopic images, exposure images). This causes problems for physicians to be able to make accurate diagnoses and perform the treatment effectively; irrespective of skill and technical excellence. The quality of the images required by physicians requires two elements: signal clarity and a low noise level, two elements that exhibit a trade‐off relationship. For example, if the guide wire is clearly displayed in a signal, the visibility is reduced as the noise increases.^1^, ^2^, ^3^ Conversely, if a recursive filter is used to reduce noise, the moving signal will become blurred due to a blurred image lag. This makes it necessary to ensure an appropriately clear signal and not to apply an overly aggressive recursive filter. For this reason, it is necessary to evaluate whether a particular recursive filter is applied appropriately.

For these reasons it is important for radiological technologists to evaluate the image, adjust the image to be optimum for clinical use, and maintain and manage the image quality.

Currently, a variety of devices are commercially available for angiography and other modalities for image evaluation.^4^, ^5^, ^6^ However, these phantoms commonly display problems when evaluating moving images. For example, commercially available phantoms for still images are not suitable for evaluation of moving images because the images are stationary.^4^ With phantoms for moving images, there are also problems: (1) many are designed for CT and MRI,^5^ and only a few for evaluation of moving fluoroscopic and exposure images; (2) also there are many phantoms for cardiac functioning analysis^5^ but only a few are specialized for the movement of coronary arteries; (3) many are designed for visual evaluations but few for physical evaluations; and (4) there are no devices designed for quantitative evaluations of moving image processing (especially image lags). Further, the movements made by some existing dynamic phantoms^6^ may be different from that of the target (blood vessel and other targets) when used for evaluations in clinical settings. For this reason, it is not certain that the actual motion of the coronary artery is accurately represented, and physical evaluations for an objective determination are difficult even when a visual evaluation is possible. Considering the problems described above, it is necessary to create reliable tools that can be simply used for daily observations of images and where it is possible to make adjustments to improve the image quality for clinical use.^7^ In this study, we create a dynamic phantom that enables image quality analysis and provides a visual evaluation unique to moving images.

As the subject of this study is for coronary angiography, it is necessary to reproduce the actual motion of the pulsating coronary artery. If a heart phantom that is beating at the current air pressure is used, analysis becomes difficult because the simulated blood vessel phantom added there will move irregularly. There are also problems with reproducibility and such a phantom would be unsuitable for daily use in examinations. There are two issues for special attention in creating a new phantom. First, the exact position of the simulated blood vessel in the phantom must be established, and second it must be possible to provide good reproducibility, and always be able to reproduce the same motion. Overall, a simulated blood vessel phantom that generates images on the screen that are closely similar to those in coronary angiograms is desirable. This study adopted a simple structure that enables physical and visual evaluations using rotational motion. To accommodate this, we developed a dynamic blood vessel phantom that reproduces coronary motion in two dimensions. We aimed to reproduce the pulsating motion of the coronary artery with the new phantom, and substitute the phantom for evaluations and adjustments of image processing in clinical settings. We also examined whether processed images (image lags) can be physically analyzed.

## 2 MATERIALS AND METHODS

### 2.1 Principles of the dynamic blood vessel phantom

**Figure 1:**
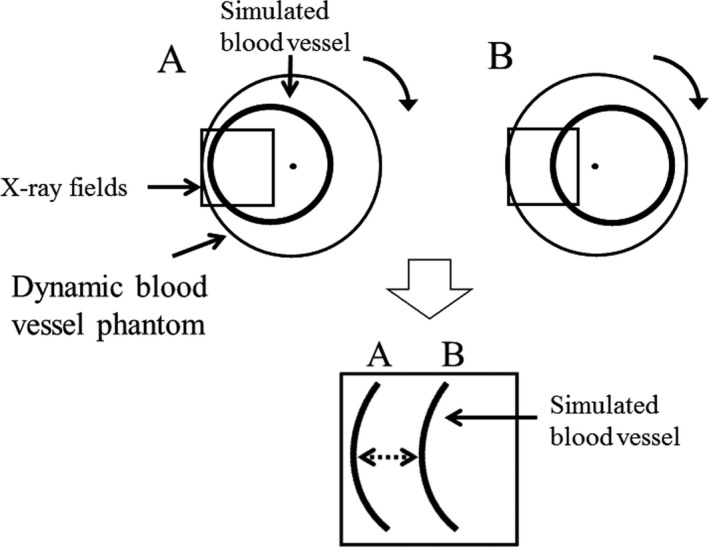
Principle of the dynamic blood vessel phantom Translational motion was reproduced by shifting the center of rotation of the phantom from the center of rotation of a circular simulated blood vessel.

Normally, the coronary artery moves three‐dimensionally, but on the projected image it shows translational motion. With the phantom we assumed that the movement of the simulated blood vessel is similar to that on the screen. However, the mechanism of the movement is very complicated and cannot be reproduced by regular piston motion. For this reason, we reproduced the pulsating translational motion by rotating the center of the round simulated blood vessel with the center shifted from the center of rotation, as suggested in Fig. 1.

For this movement, an acrylic disc with a circular aluminum wire of a smaller diameter was used to represent the simulated blood vessel. By shifting the center of rotation of the disc and observing a part of the region, we were able to reproduce the left right motion of the simulated blood vessel.

**Figure 2:**
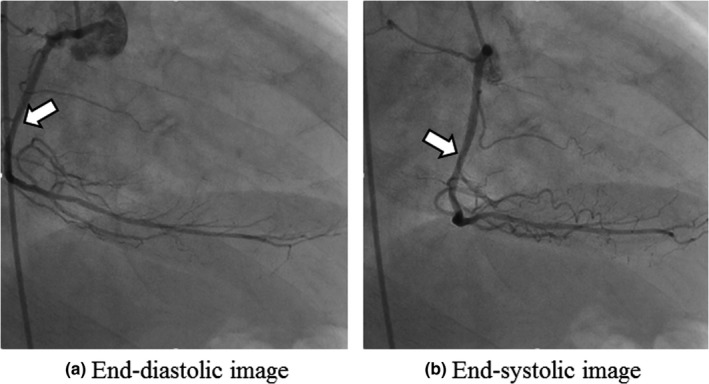
Normal right coronary angiograms modeled for the phantom (RAO 30°). RAO, right anterior oblique.The white arrows indicate the measurement point of the coronary artery.

Here, we assumed a normal (unaffected by any ailment) coronary angiogram with a heart rate of 60 bpm (beats per minute) and a left ventricular ejection fraction of above 60%, and recorded photographs of the simulated blood vessel at 15 frames per second (fps) with an angiograph. The imaging angles of the coronary angiography of clinical images used in our hospital were as follows: Five directions for the left coronary angiography (RAO 30°/CAUD 20°, RAO 30°/CRA 20°, AP/CRA 20°, LAO 30°/CRA 20°, LAO 40°/CAUD 30°) and 3 for the right (LAO 60°, LAO 30°/CRA 20°, RAO 30°). We selected a right anterior oblique angle of (RAO) 30° for the imaging angle of the right coronary angiography with the movement distance the largest among these. Figure 2 shows clinical images used for reference. Here, LAO, CAUD, and CRA stand for left anterior oblique angle, caudal, and cranial, respectively.

**Figure 3:**
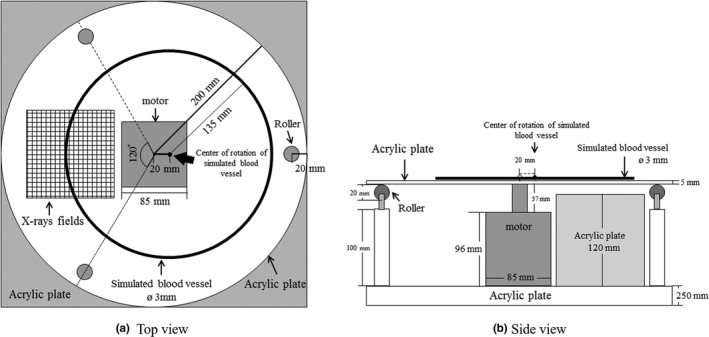
Schematic outline of the phantom.

For the dynamic blood vessel phantom, we used a 60 rpm motor so that one rotation is equivalent to one heartbeat, reproducing normal coronary angiographic images with a heart rate of 60 bpm. For the base of the phantom, we used a 25 mm thick acrylic plate with a motor (rotor) at the center. On the rotor we placed a 200 mm radius 5 mm thick acrylic disc with an aluminum wire in a perfect circle, radius 135 mm, which is assumed to simulate the blood vessel, both on the acrylic base disc with the motor. Assuming a coronary artery, we set the diameter of the simulated blood vessel to 3 mm. Also, on the base disc we placed an acrylic cylinder with rollers on top at 120‐degree intervals, positioned at 20 mm inward from the outer edge of the acrylic disc, so that distortion of the rotational motion is reduced when the acrylic disc rotates on the rollers. Further, we added a 120 mm thick acrylic plate between the acrylic disc (5 mm) and the acrylic base plate (25 mm), making the total thickness of acrylic plate 150 mm, to make the condition of photographing the dynamic blood vessel phantom close to that of the thickness of the human body. By making the thickness of the acrylic plate closer to the thickness of the human body, it became possible to evaluate moving images under clinical imaging conditions. Figure 3 shows the design of the dynamic blood vessel phantom.

### 2.2 Evaluation of motion

**Figure 4:**
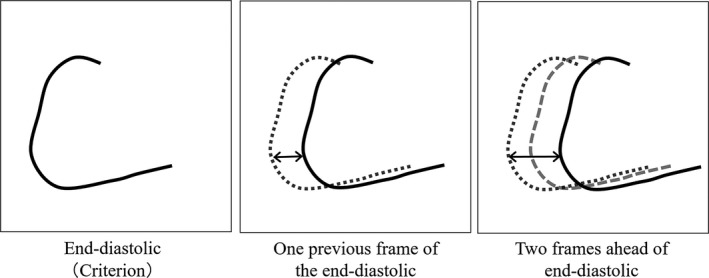
Measurement principles for the distances of movement of the right coronary angiograms from the previous image.

We compared the motion of a coronary artery with the coronary angiography and that of the simulated blood vessel of the dynamic blood vessel phantom. First, we measured the distance which the coronary artery moved on the screen using clinical coronary angiography. The measurement was at the bifurcation of the right coronary artery trunk and the acute marginal (AM) branch. Assuming the maximum diastolic period as the reference criterion, the distances which the coronary artery moved from the reference criterion were measured in every frame. The distances that the coronary artery had moved in the plane were calculated from the number of pixels it was displaced, using ImageJ software.^8^ Figure 4 shows the measurement method. Further, we decided to evaluate these distances for three heartbeats of the person (clinical angiogram) we refer to.

**Figure 5:**
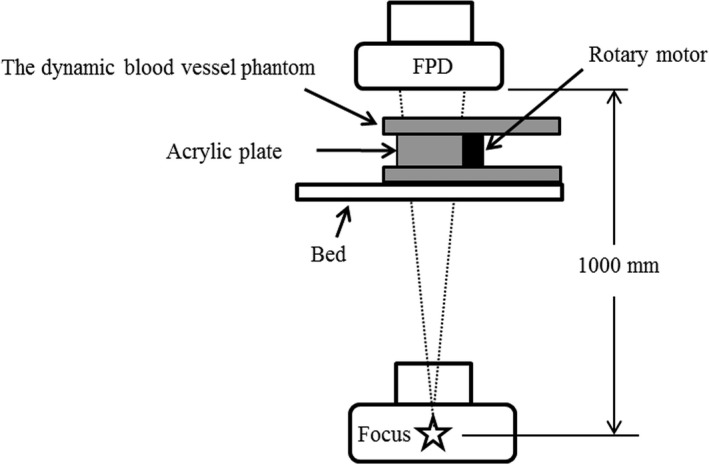
Plan of placement of the apparatus for angiography and the dynamic blood vessel phantom.

Fluoroscopy of the dynamic blood vessel phantom was performed with an apparatus for angiography (Allura Clarity FD20/20, Philips). We used a locally developed coronary angiography program clinically (tube voltage and tube current: Auto, fluoroscope: 7.5 p/ s), with a visual field size of 8 inches. For the geometry of the fluoroscopic photography, the Source Image Distance (SID) was set to 1000 mm, which is closely equivalent to that in the clinical coronary angiography, and the position of the simulated blood vessel was set as the irradiation reference criterion for the patient. A schematic diagram representing this setup is shown in Fig. 5. Assuming the image of the simulated blood vessel of the obtained dynamic vascular phantom image at the outermost position as a reference image, we measured the distances which the simulated blood vessel moved towards the innermost direction in each frame. Then, the reproducibility was confirmed by comparing the motion of the coronary artery of the clinical image with that of the simulated blood vessel of the dynamic blood vessel phantom.

### 2.3 Evaluation of image lag

**Figure 6:**
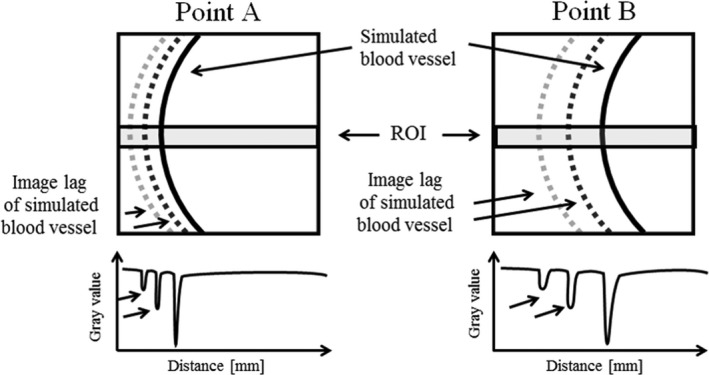
Method used in the image analysis to evaluate the image lag A profile curve was drawn by setting the ROI to the center of the image. ROI, Region of Interest.

We evaluated the fluoroscopic images and image lags obtained in Experiment B, with the measurement method as shown in Fig 6. The measurement points were set at two positions with different motions. One, at Point A was for the timing when the simulated blood vessel was at the outermost position, and the other, Point B was the timing when the vessel was moving from the outer to the inner position, the 3^rd^ frame from Point A. For the measurements, we drew a profile at the center of the image using ImageJ and setting the Region of Interest (ROI) as a width of 150 × 1000 pixels. To prevent the simulated blood vessel, which is a digital signal, from being buried in noise, we provided the ROI with some allowance. From the profile of Points A and B, the presence of image lags was determined.

For the measurements, we used the photographs of four consecutive images, with the earliest image as Frame 0, the next as Frame 1, and the last as Frame 3. In the quantitative evaluation of the image lags, we set the position where there is a signal from the profile of Frame 1 to the ROI, and calculated the average pixel number it covers. Similarly, we calculated the average pixel number covered at the same ROI position for the frames before and after this. Next, we calculated the differences in the average pixel numbers of Frame 0 and each of the following frames. Finally, the difference between Frame 0 and Frame 1 was normalized to 1.0. The differences between Frame 0 and Frame 2, and Frame 0 and Frame 3 are considered as image lags because they are not signals, although the difference between Frame 0 and Frame 1 is a signal.

The developed phantom allowed us to obtain fluoroscopic moving images by changing the pulse rate from 15 f/s to 7.5 f/s under conditions with many different visual image lags (different parameters such as those for image processing). Analysis was performed using the dynamic blood vessel phantom in the manner described above, and the ratio of the image lags was determined.

### 2.4 Evaluation of image lags using the recursive filters with different intensities

Further, a quantitative evaluation of the image lags was performed using moving images with the intensity of the recursive filters changed. In the quantitative evaluation we used moving images with the intensity of the recursive filters changed to six levels (A: none, B: weak, C: somewhat weak, D: intermediate, E: somewhat strong, F: strong). In the analysis, the same frames of the six kinds of moving images were extracted as still images and profiles were drawn.

The root mean square granularity (RMS) was calculated by the following equation:

(1) $$\text{RMS}=\sqrt{\frac{1}{n}\sum_{i=1}^{n}{\left(D_{i}-\bar{D}\right)}^{2}}$$

n= the number of data, *Di* = density data of each image, $\bar{D}=$ mean value of the image density in the data. We extracted frames from the obtained moving images as still images, and visually evaluated them with 10 radiological technologists to determine the applicability for clinical use.

## 3 RESULTS

### 3.1 Principles of the Phantom

**Figure 7:**
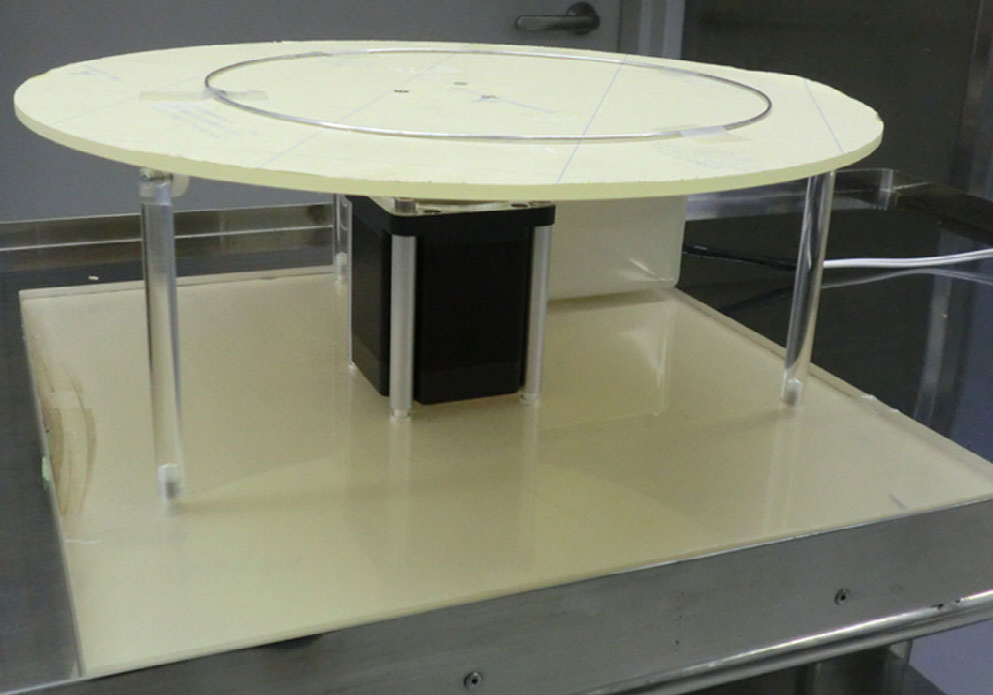
Photograph of the dynamic blood vessel phantom developed.

Figure 7 shows a photograph of the dynamic blood vessel phantom developed here.

### 3.2 Evaluation of motion

**Figure 8:**
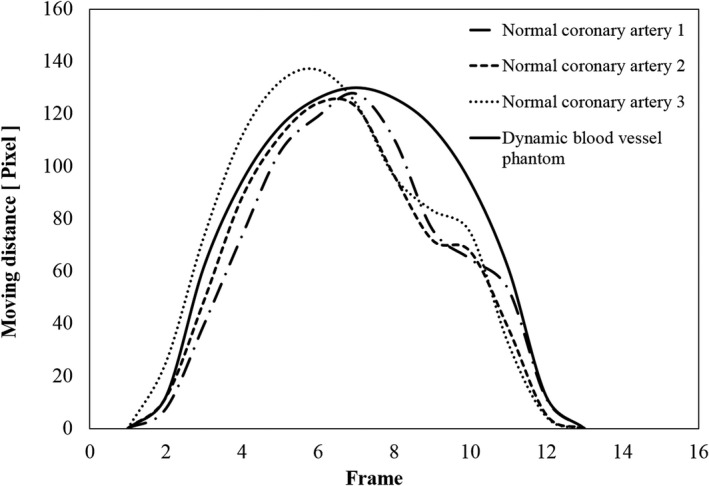
Plot of motion of blood vessel phantom and a normal coronary artery.

**Figure 9:**
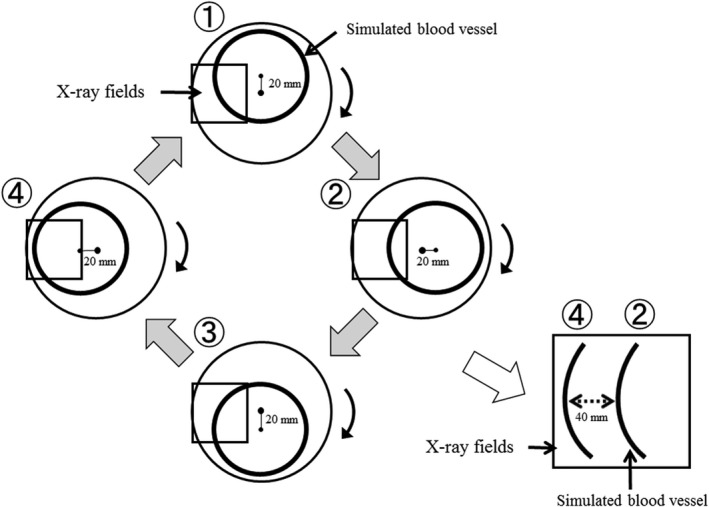
Details of why normal coronary artery and the dynamic blood vessel phantom show similar motion Translational motion was reproduced by shifting the center of rotation of the acrylic disc and that of the simulated blood vessel.

Figure 8 shows the results of the analysis of the motion changes of the simulated blood vessel on the dynamic phantom and of a normal coronary artery. The figure shows that the waveform of the normal coronary artery moved by 130 pixels (about 25 mm) in the waveforms of three heartbeats. During fluoroscopy, when a part of the area of the phantom (irradiation field) with the geometry shown in Fig. 5 was observed, the coronary artery appeared as translated 40 mm because we shifted the center of rotation of the disc and the simulated blood vessel by 20 mm (Fig. 9).

Further, the graph shows that the simulated blood vessels can reproduce a movement that is very similar to that of normal coronary arteries. However, there was a disparity between the movement position of the normal coronary artery and the simulated blood vessel when changing from the systole to part of the blood flow.

### 3.3 Evaluation of image lag

**Figure 10:**
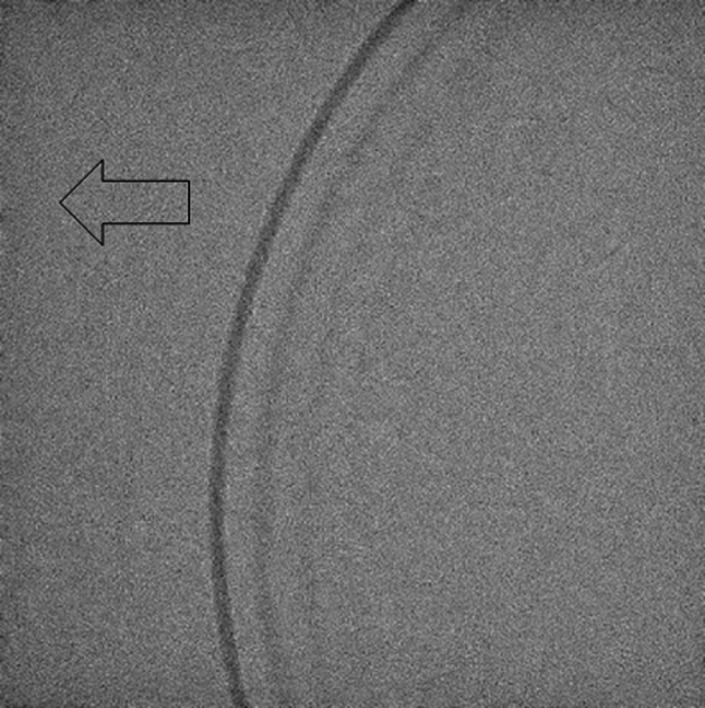
Fluoroscopic image of the dynamic phantom (Point A).

**Figure 11:**
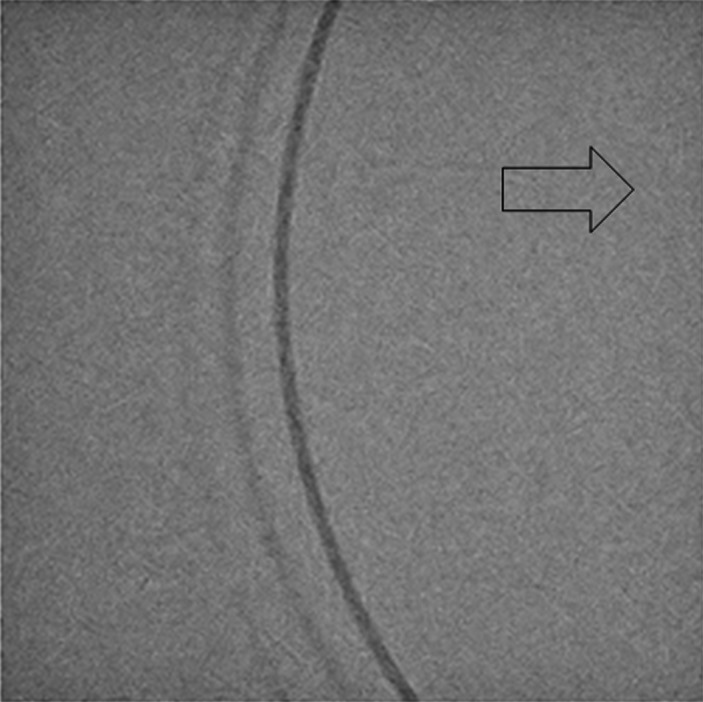
Fluoroscopic image of the dynamic phantom (Point B).

**Figure 12:**
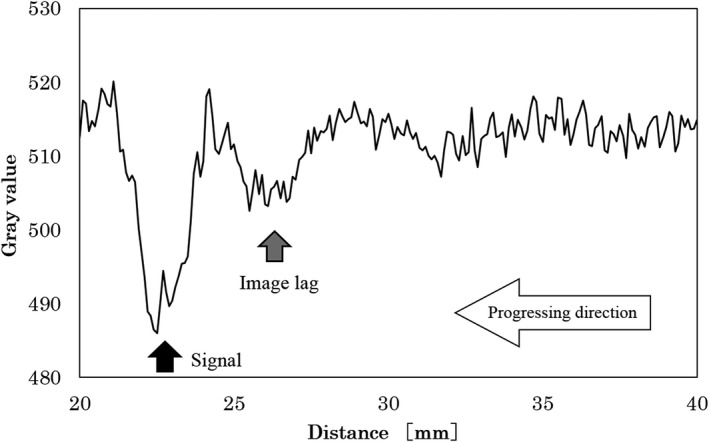
Profile curve at Point A.

**Figure 13:**
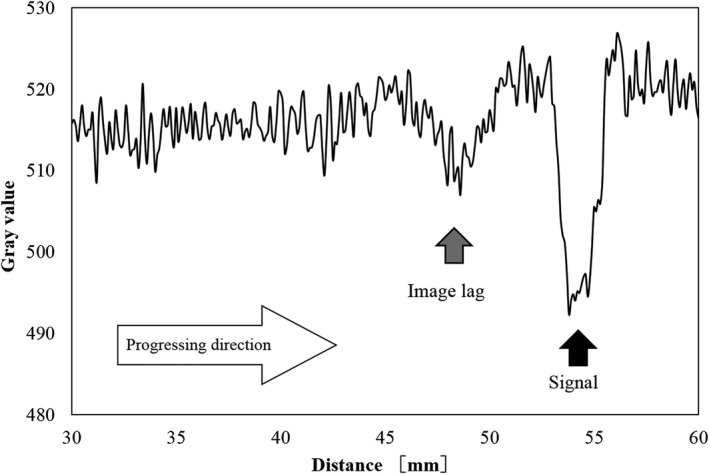
Profile curve at Point B.

**Figure 14:**
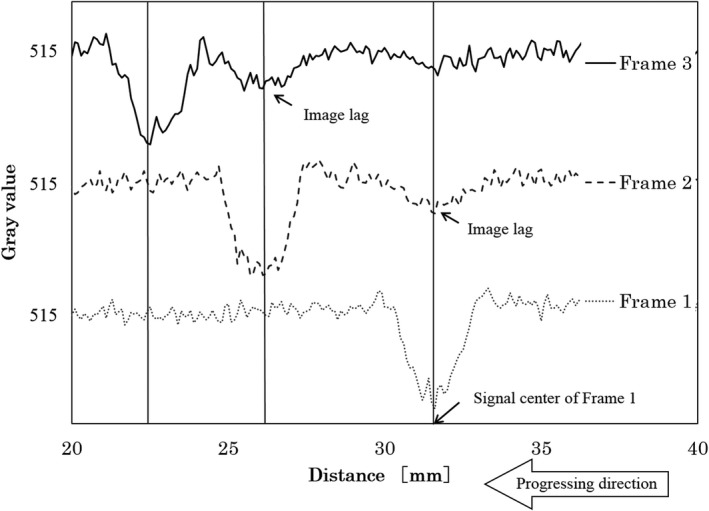
Plot of profile curves of three consecutive images at Point A.

**Figure 15:**
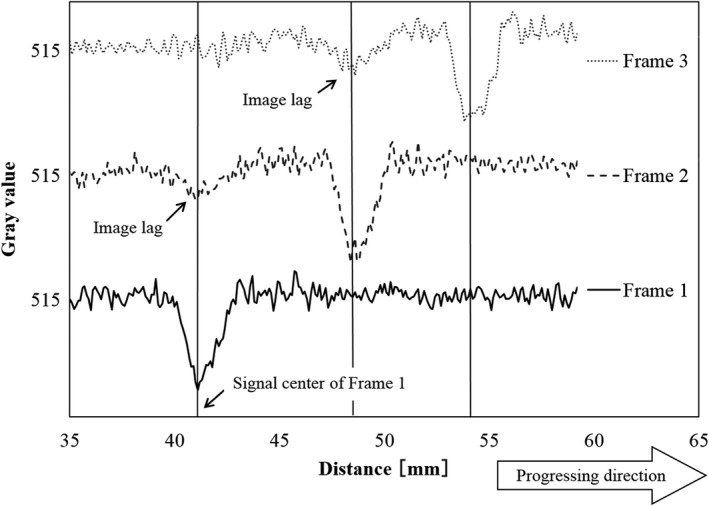
Plot of profile curves of three consecutive images at Point B.

There were image lags in the fluoroscopic image. Figures 10 and 11 shows the fluoroscopic images of the dynamic blood vessel phantom. As shown in Figs. 10 and 11, there were image lags that formed arcs similar to two simulated blood vessels after the simulated blood vessel moved in the fluoroscopic images. The image lags appear at the position where the signal appeared in the previous image. Further, we can observe that an image lag of an (first) image lag is also generated. Therefore, the first residual image, closest to the simulated blood vessel, showed up most clearly, while secondary residual images were less distinct. Figures 12 and 13 show the results of evaluations of image lags by drawing profile using ImageJ. At the position of the simulated blood vessel, the pixel gray level became the smallest. A signal appearing at a position where there is no simulated blood vessel is considered as an image lag because it is not a real signal (false signal). Figures 14 and 15 show graphs of the profile of three consecutive images in the signals of Point A and Point B. With one previous image (Signal center of Frame 1), there was a simulated blood vessel at the position of the false signal. This makes it possible to determine that the false signal is an image lag generated from the signal of the simulated blood vessel.

**Figure 16:**
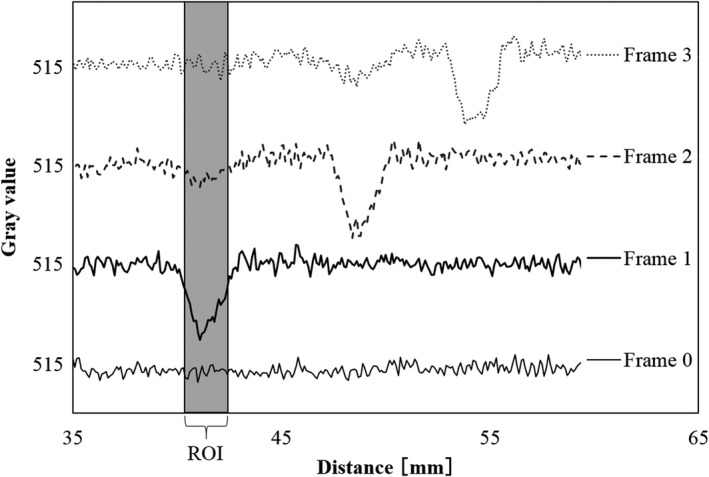
Image lag measurements using the profile curves (Point B).

**Figure 17:**
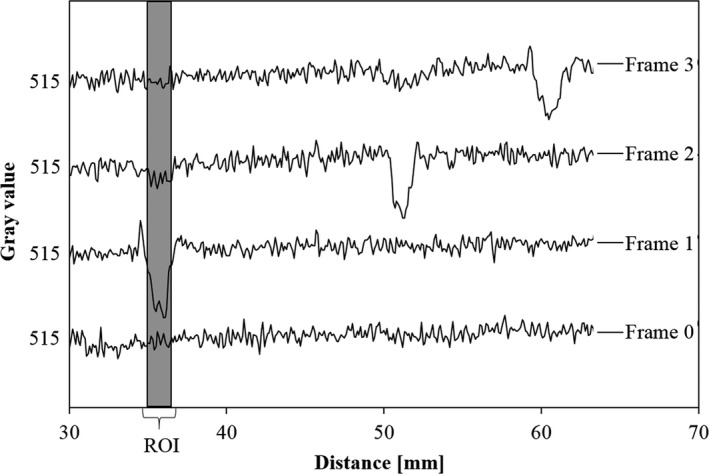
Profile curves of the dynamic blood vessel phantom using different image processing parameters.

Profiles of four consecutive images are shown in Fig. 16. The results of a quantitative evaluation based on Fig. 16 are shown in Table 1. Setting Frame 1 to 1.0, we calculated the average pixel value of the image lag. As a result, we were able to measure the amount of the image lags and the results are as follows: 0.24 for the same position of the next image (Frame 2), and 0.03 after 2 images (Frame 3). Figure 17 and Table 2 show the results obtained from the fluoroscopic images obtained under conditions with many visual image lags. Compared to the results shown in Table 1, image lag ratios increased from 0.24 to 0.29.

**Table 1:** Quantitative evaluation of image lags (Point B).

	Average pixel number	Difference from Frame 0	Normalized difference in pixel values of Frame 0 and Frame 1	
Frame 0	516.20	–	–	–
Frame 1	498.05	18.15	1.00	Signal
Frame 2	511.79	4.42	0.24	Image lag
Frame 3	515.63	0.57	0.03	Image lag

**Table 2:** Quantitative evaluation of image lags (different image processing parameters).

	Average pixel number	Difference from Frame 0	Normalized difference in pixel values of Frame 0 and Frame 1	
Frame 0	512.70	–	–	–
Frame 1	500.20	12.50	1.00	Signal
Frame 2	509.13	3.57	0.29	Image lag
Frame 3	512.13	0.57	0.05	Image lag

### 3.4 Evaluation of image lags using the recursive filters with different intensities

**Figure 18:**
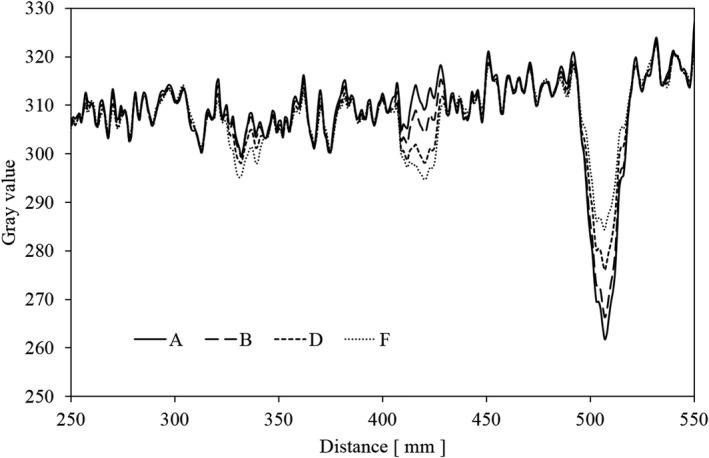
Profile curve of moving image with various intensities of the recursive filter.

Figure 18 shows the profiles of the moving A, B, D, and F images. Then, the image lags were quantified (Lag 1, Lag 2) based on the simulated blood vessels (signals) of each moving image from the gray value of the profile. Table 3 shows the results of the quantification. Figure 18 shows no detectable peak for the residual image when no recursive filter is used (A), and that the contrast between the simulated blood vessel and the background is largest in this case. In the F image, with the strongest recursive filter intensity, the noise component was somewhat smaller than in A, and the difference between the background and the simulated blood vessel (signal) gray value was smaller. This also appears in the numerical value as shown in Table 3.

**Table 3:** Relationship between graininess (noise), simulated blood vessel, and image lag in the fluoroscopic images.

Fluoroscopic images	A	B	C	D	E	F
RMS（SD）	21.6	19.8	18.2	16.8	15.8	15.0
Signal [%]	100.0	91.4	89.5	89.1	88.9	88.6
Lag 1 [%]	0.0	12.1	22.9	34.1	45.5	57.1
Lag 2 [%]	0.0	1.7	4.0	8.5	14.9	23.1

RMS, root mean square granularity.

**Figure 19:**
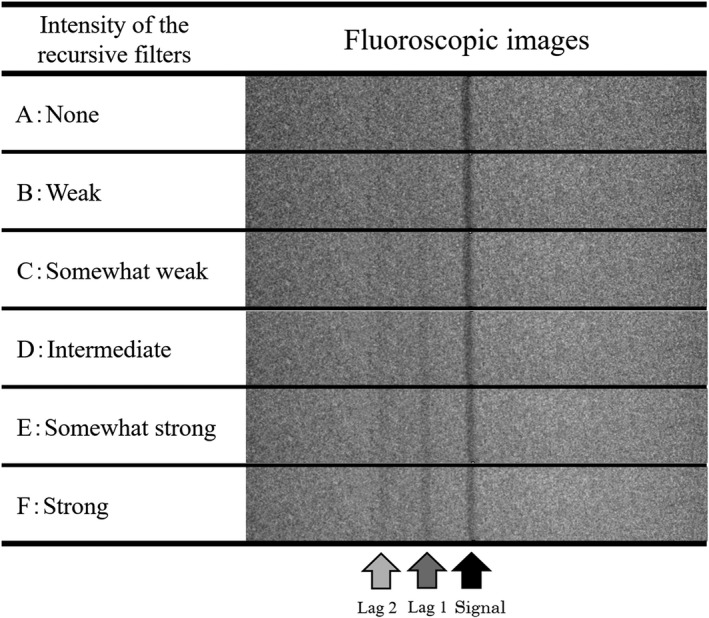
Still image cut out from the moving image with various intensities of the recursive filter.

The noise showing graininess shows that the numerical value decreases as the intensity of the recursive filter increases from A to F. The simulated blood vessel (signal) was 100% in A, and decreased as the intensities of the recursive filter increased from B to F. However, there was no image lag in A, but the residual signal intensity gradually increases from B to F in both Lag 1 and Lag 2. Comparing Lag 1 and Lag 2, it is clear that the image lag in Lag 1, which appeared in a position close to the signal in the moving image with the recursive filter, is larger. The same result as in the quantitative evaluation was obtained also when we visually evaluated the moving images separately. In the visual evaluation, seven radiologic technologists evaluated the movement of images from A to B as suitable for clinical use, and three evaluated A to C. Figure 19 shows a still image of the evaluated moving images. With still images, A to F are applicable for clinical use because image lags will not be generated even when the intensity of the recursive filter is changed.

## 4 DISCUSSION

Currently, there are few commercially available phantoms for moving image evaluations. Further, in the phantom used to evaluate moving images, wires rotate like the hands of a clock, and so show motion different from that of actual blood vessels.^9^ For this reason, there is presently no phantom that reproduces the motion of clinical coronary arteries, and which enables quantitative evaluations. With the dynamic vascular phantom developed in the present study, the motion of the simulated blood vessel is constant because it uses rotational motion while reproducing the motion of the coronary artery. It was found that the ratio of image lag increased from 0.24 to 0.29 by changing the pulse rate from 15 f/s to 7.5 f/s under conditions with many different visual image lags. This corresponds to the results of visual evaluation, suggesting that quantitative evaluations of moving image may make it easier to compare the differences in the effect of image processing.

In summary, when the intensity of the recursive filter is weak, the difference between the gray value of the simulated blood vessel (signal) and the background decreases slightly, a small image lag appears, and the noise level is high. When the intensity of the recursive filter is strong, the difference between the gray value of the background and the simulated blood vessel (signal) decreases, a large image lag appears, and the noise level decreases. It is advantageous to increase the intensity of the recursive filter to reduce the noise of the moving images. The degree to which we can increase the recursive filter strength is limited by the appearance of residual images and the loss of contrast between the simulated vessel and the background at higher filter strengths. Therefore, it is necessary to determine a maximum intensity of the recursive filter within the acceptable range for the image lag.

For this reason, we performed a visual evaluation of the moving and still images from A to F (Fig. 19) to identify an acceptable range of image lags, evaluated by 10 medical radiologists who are engaged in cardiac catheter examinations. Here, seven radiologists evaluated the moving images suitable for clinical use as A to B, and three evaluated it as A to C. In the still image, five radiologists evaluated it as A and B, and five as A to C. The reason for the differences in the evaluations for the moving and still images may be because the impression of the image lag becomes stronger than the noise in the moving image, and the image lag becomes less visible due to the noise in the still image as the simulated blood vessel is a signal of high contrast. This could suggest that in the visual evaluation, and when evaluating the moving image with the still image, it may become impossible to establish a single determination of the qualities of the evaluation something that is generally considered necessary for the image processing.

As discussed above, this dynamic blood vessel phantom makes it possible to evaluate the differences in image quality quantitatively even when different recursive filters are used.

Traditionally, when we adjust image quality in clinical settings, we have to do this gradually to obtain the wished for quality while utilizing the image processing in a manner recommended by the manufacturer. However, by using the dynamic blood vessel phantom developed in this study, a clinical evaluation during the CAG becomes unnecessary. Further, as the phantom reproduces the motion of the coronary artery, it enables conducting CAG and PCI in an optimum state where images have been processed and adjusted to the extent that physicians require. The results strongly suggest that physicians and medical staff in charge of CAG and PCI will be able to conduct CAG with confidence using properly processed images.

## 5 CONCLUSIONS

In this study, we developed a dynamic blood vessel phantom that reproduces the motion of the coronary artery two‐dimensionally using rotational motion. Because this phantom has signals and image lags at the same position, a quantitative evaluation of the image lags is possible. Overall, in the evaluation of image processing with the angiograph, image processing evaluations (image lags) which are performed in clinical settings can be substituted by a dynamic blood vessel phantom. Further studies are necessary to improve the phantom developed here to serve as an evaluation tool to manage clinical images on a daily basis.

## Conflict of Interest

The authors declare that they have no conflicts of interest to report.
